# Nerve Wrap for Local Delivery of FK506/Tacrolimus Accelerates Nerve Regeneration

**DOI:** 10.3390/ijms25020847

**Published:** 2024-01-10

**Authors:** Bo Xiao, Firuz Feturi, An-Jey A. Su, Yolandi Van der Merwe, Joshua M. Barnett, Kayvon Jabbari, Neil J. Khatter, Bing Li, Evan B. Katzel, Raman Venkataramanan, Mario G. Solari, William R. Wagner, Michael B. Steketee, Daniel J. Simons, Kia M. Washington

**Affiliations:** 1 Department of Plastic Surgery, University of Pittsburgh School of Medicine, Veterans Administration Healthcare System, Pittsburgh, PA 15213, USA; xiaobo@fmmu.edu.cn (B.X.); fgf3@pitt.edu (F.F.);; 2 Department of Surgery, Division of Plastic Surgery, University of Colorado Anschutz Medical Campus, Aurora, CO 80045, USA; 3 Department of Neurobiology, University of Pittsburgh School of Medicine, Pittsburgh, PA 15213, USA; 4 Department of Ophthalmology, University of California, San Diego, CA 90095, USA; 5 University of Pittsburgh School of Pharmacy, Pittsburgh, PA 15213, USA; 6 McGowan Institute for Regenerative Medicine, Pittsburgh, PA 15219, USA; wagnerwr@upmc.edu (W.R.W.); cortex@pitt.edu (D.J.S.)

**Keywords:** peripheral nerve injury, tacrolimus, neuroregeneration, immunology, infraorbital nerve, biopolymer

## Abstract

Peripheral nerve injuries (PNIs) occur frequently and can lead to devastating and permanent sensory and motor function disabilities. Systemic tacrolimus (FK506) administration has been shown to hasten recovery and improve functional outcomes after PNI repair. Unfortunately, high systemic levels of FK506 can result in adverse side effects. The localized administration of FK506 could provide the neuroregenerative benefits of FK506 while avoiding systemic, off-target side effects. This study investigates the utility of a novel FK506-impregnated polyester urethane urea (PEUU) nerve wrap to treat PNI in a previously validated rat infraorbital nerve (ION) transection and repair model. ION function was assessed by microelectrode recordings of trigeminal ganglion cells responding to controlled vibrissae deflections in ION-transected and -repaired animals, with and without the nerve wrap. Peristimulus time histograms (PSTHs) having 1 ms bins were constructed from spike times of individual single units. Responses to stimulus onsets (ON responses) were calculated during a 20 ms period beginning 1 ms after deflection onset; this epoch captures the initial, transient phase of the whisker-evoked response. Compared to no-wrap controls, rats with PEUU-FK506 wraps functionally recovered earlier, displaying larger response magnitudes. With nerve wrap treatment, FK506 blood levels up to six weeks were measured nearly at the limit of quantification (LOQ ≥ 2.0 ng/mL); whereas the drug concentrations within the ION and muscle were much higher, demonstrating the local delivery of FK506 to treat PNI. An immunohistological assessment of ION showed increased myelin expression for animals assigned to neurorrhaphy with PEUU-FK506 treatment compared to untreated or systemic-FK506-treated animals, suggesting that improved PNI outcomes using PEUU-FK506 is mediated by the modulation of Schwann cell activity.

## 1. Introduction

The poor outcomes following treatment of peripheral nerve injury (PNI) are a frequent clinical challenge that affects the overall quality of life for patients across the world. The most common peripheral neuropathies are traumatic nerve injuries, representing nearly 5% of patients admitted to Level I trauma centers [1]. Severe neuropathic pain and disability contribute to a poor quality of life in those with traumatic neuropathies [2]. While nerve regeneration in the central nervous system (CNS) is very limited, peripheral nerves can regenerate their axons if the injury is relatively small, though larger injuries necessitate surgical intervention [3,4]. Surgical treatment of PNI such as end-to-end repair and nerve grafting provide some return of function, but many patients exhibit incomplete recovery. Follow-up reports of median and ulnar nerve surgical repair found that 51.6% of patients achieved satisfactory motor recovery, while only 42.6% experienced satisfactory sensory recovery, resulting in substantial disability [5,6,7]. The surgical repair technique appears to play a minor role in functional outcomes following PNI treatment [8]. Instead, nerve regeneration properties may play a larger role in recovery. Canonical descriptions of peripheral nerve recovery after PNI begins with (1) Wallerian degeneration and concurrent distal stump demyelination due to the loss of Schwann cell contact, followed by (2) a robust axon regenerative program including growth cone formation and axon outgrowth that is promoted by trophic factors associated with dedifferentiated Schwann cells, and, finally (3) end-organ reinnervate and axon re-myelination after Schwann cells regain contact and re-differentiate into myelinating cells [9,10]. Expression of intrinsic growth factors and neurotropic factors needed for axonal regeneration is negatively correlated to increased time and transection gap distance [11,12,13,14]. In cases where large transection gap distances between proximal and distal nerve segments persist for an extended duration, neurons undergo cellular apoptosis, curtailing regeneration [8]. Thus, novel neuroregenerative strategies for increasing the rate of peripheral nerve regeneration are needed to improve outcomes in these PNI patients.

FK506 (Tacrolimus) along with rapamycin are two of the most widely used immunosuppressive drugs in solid organ transplantation (SOT) and vascularized composite allotransplantation (VCA) to improve graft survival and reduce rejection [15]. FK506 is an FDA-approved immunosuppressant that acts as a potent inhibitor of calcineurin to modulate T-cell-mediated allograft rejection [16,17]. Beyond its immunosuppressive effects, FK506 mediates neurotrophic and neuroprotective effects via several mechanisms [18,19,20,21,22]. Experimentally, FK506 significantly increases both the rate of axonal regeneration and myelinated nerve fiber density following peripheral nerve transection and repair [23,24,25]. Overall, systemic FK506 administration accelerates nerve recovery and improves outcomes after nerve injury and repair [26,27,28,29,30,31]. However, systemic immunosuppression is responsible for numerous poorly tolerated, global side effects that can lead to organ failure [32,33,34,35,36,37].

Since 1998, more than 200 VCA procedures have been successfully performed [38]. VCAs such as hand, face, uterus, or abdominal wall transplants are unique from SOTs because of their heterogenous tissue composition that may include skin, muscle, vessels, tendon, nerve, lymph nodes, cartilage, bone, and bone marrow. Notably, skin has been shown to be the most immunogenic constituent of certain VCAs, mandating long-term immunosuppression for graft survival [39]. Unlike SOTs, VCAs uniquely offer opportunities for the local delivery of immunosuppressants directly to the graft, potentially reducing the systemic exposure and global collateral of end-organ adverse effects. Additionally, the transplantation of a whole limb allograft reportedly elicits a lower immune response compared to that of individual tissue components in the form of vascularized or non-vascularized grafts [39]. Thus, site-specific graft immunosuppression could minimize the overall dosing, frequency, and duration of systemic immunosuppression. Another potential benefit includes the concomitant reduction of the total number of systemic drugs needed to ensure or improve graft survival [15]. As such, studies have focused on various carriers for the delivery of FK506 [40,41,42,43]. Initial efforts to limit systemic toxicity by the local delivery of FK506 to treat PNI [44,45] were complicated by the need for a controlled and sustained release of FK506 [46]. More recently, engineered delivery solutions are reported to address these issues successfully in treating sciatic nerve models of PNI [15,40,43]. Studies have reported decreased neuroma formation and improved nerve healing when bridging small gaps with nerve wraps during repair [47,48,49]. Previously, we demonstrated a flexible polyester urethane urea (PEUU) elastomer blended with FK506 (PEUU-FK506) consistently releases FK506 over 14 days with positive effects on axon outgrowth in vitro or in a rat optic nerve crush model [50]. In this study, we investigate the use of PEUU-FK506 as a new pre-clinical therapy targeted at the enhanced recovery of sensory function in damaged peripheral nerves.

To test the bioactivity of PEUU-FK506 nerve wrap in vivo, we examined its effects when used in repairing infraorbital nerve (ION) transection in rat. In rats, mechanotransduction of tactile information is detected by facial vibrissae (whiskers) [51,52]. The signal travels through several synaptic stations from the primary sensory nerves (branches of the infraorbital nerve) to the somatosensory cortex. Previously, we reported on the response properties of trigeminal ganglion cells evoked by controlled whisker stimulation following ION transection and microsurgical repair in adult rats [53]. Here, we use the rodent whisker/trigeminal system model to assess the effect of PEUU-FK506 on mechanoreceptive afferents [51,52,54]. Additionally, we measured the concentration of FK506 in the blood serum, ION, and distal tissues of subjects treated with the PEUU-based wraps to quantify the level of systemic FK506 produced by these wraps in vivo. Lastly, we performed an immunohistochemical analysis of the recovered tissue to assess the neurotrophic properties of PEUU-FK506 upon myelin and axonal neurofilament dynamics in the recovery of a purely sensory nerve.

## 2. Results

### 2.1. Concentration of FK506 in PEUU-FK506 Wrap, Serum, and Tissue Samples

**Figure 1:**
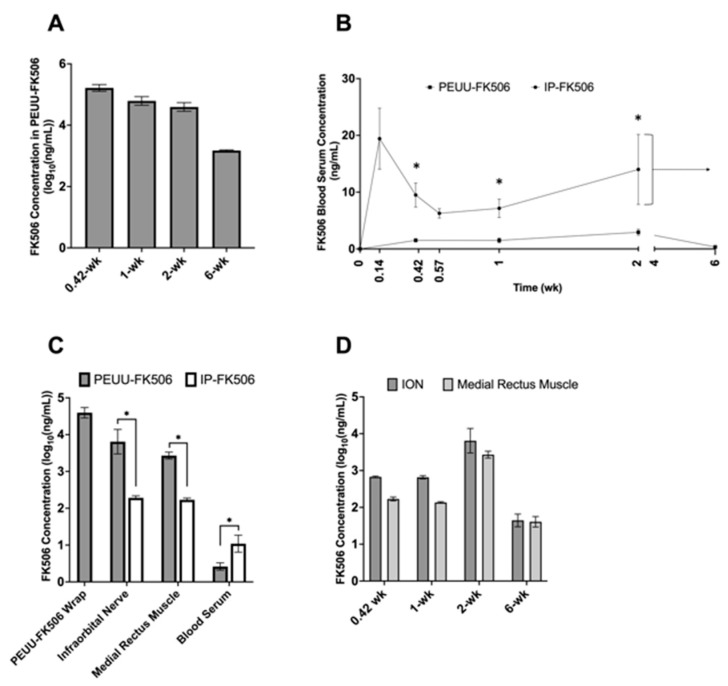
(**A**) FK506 concentration within PEUU-FK506 wrap over six weeks shows sustained release of drug. FK506 concentration represented as a log_10_ transformation of mean ± SEM. (**B**) Blood serum levels of FK506 are lower for PEUU-FK506-treated animals versus IP-FK506-treated animals. FK506 blood concentration for animals (*n* = 3–6/group/time point) treated with PEUU-FK506 (black line) and animals treated with IP-FK506 (dotted line) over six weeks. Significant difference in mean FK506 blood serum concentration between groups denoted as * with *p* < 0.05 using Mann–Whitney test. Beyond two weeks, IP-treated animals reached steady state FK506 blood concentrations represented as mean concentration (dotted arrow) and standard error of means (bracket). Blood concentration values represented as mean ± SEM. (**C**) PEUU-FK506 delivers higher drug concentrations to the infraorbital nerve when compared to IP-FK506 treatment at two weeks postoperatively. Comparative FK506 concentrations within the infraorbital nerve, medial rectus muscle, blood serum, and PEUU-FK506 wrap among animals (*n* = 3–6/group/time point) treated with IP-FK506 2 mg/kg/day or PEUU-FK506 infraorbital nerve wrap for two weeks. FK506 concentration represented as a log_10_ transformation of mean ± SEM. (**D**) PEUU-FK506 delivers drug in higher concentrations locally versus in distal tissues. FK506 concentrations in the infraorbital nerve and medial rectus muscle over time among PEUU-FK506-treated animals (*n* = 3–6/group/time point). FK506 concentration represented as a log_10_ transformation of mean ± SEM.

The FK506 concentration was quantified in the PEUU-FK506 wrap, ION, medial rectus muscle, and peripheral blood serum up to six weeks postoperatively for the 12 rats treated with PEUU-FK506 wrap. Six additional rats were given daily intraperitoneal (IP) injections of 2.2 mg/kg FK506. These IP-FK506 treated rats were assayed for FK506 concentration within the ION, medial rectus muscle, and blood serum. Each nerve wrap contained 0.4 mg of FK506 prior to implantation. The PEUU-FK506 wrap exhibited sustained release of drug up to six weeks postoperatively (Figure 1A). Blood serum concentrations of FK506 were lower in the PEUU-FK506 group compared to the IP-FK506 group during this time (Figure 1B). At 0.4 weeks, the FK506 blood serum concentration for PEUU-FK506 and IP-FK506 groups measured 1.52 ± 0.32 ng/mL and 9.5 ± 2.1 ng/mL (mean ± SEM, Mann–Whitney test, *p* = 0.03), respectively. At one week, the blood serum level of FK506 remained significantly lower for PEUU-FK506-treated animals compared to the IP-FK506 group (1.50 ± 0.41 ng/mL vs. 7.15 ± 1.62 ng/mL; mean ± SEM, Mann–Whitney test, *p* = 0.02). At two weeks postoperatively, the blood serum FK506 concentration measured 2.93 ± 0.54 ng/mL (mean ± SEM) for PEUU-FK506-treated animals, while the level for daily IP-FK506-treated animals was 14.0 ± 6.16 ng/mL (mean ± SEM). The systemic concentration of FK506 after two weeks of treatment was significantly lower in the PEUU-FK506 animals, measuring approximately ~78% lower than IP-FK506-treated animals (Mann–Whitney test, *p* = 0.05).

We found that, at two weeks postoperatively, the FK506 concentration in the ION of PEUU-FK506-treated animals was 20.46 ± 9.31 mg/kg compared to 0.19 ± 0.03 mg/kg in IP-FK506-treated animals (mean ± SEM, Mann–Whitney test, *p* = 0.048), representing significantly greater drug enrichment within the nerve with wrap-based treatment (Figure 1C). The measured FK506 concentration within the PEUU-FK506 wrap itself was 51.27 mg/kg at 2-weeks. Lastly, PEUU-FK506 treatment delivered higher concentrations of the drug locally, within the ION versus in distal tissues up to six weeks postoperatively (Figure 1D).

### 2.2. Trigeminal Ganglion Cell Recordings

**Figure 2:**
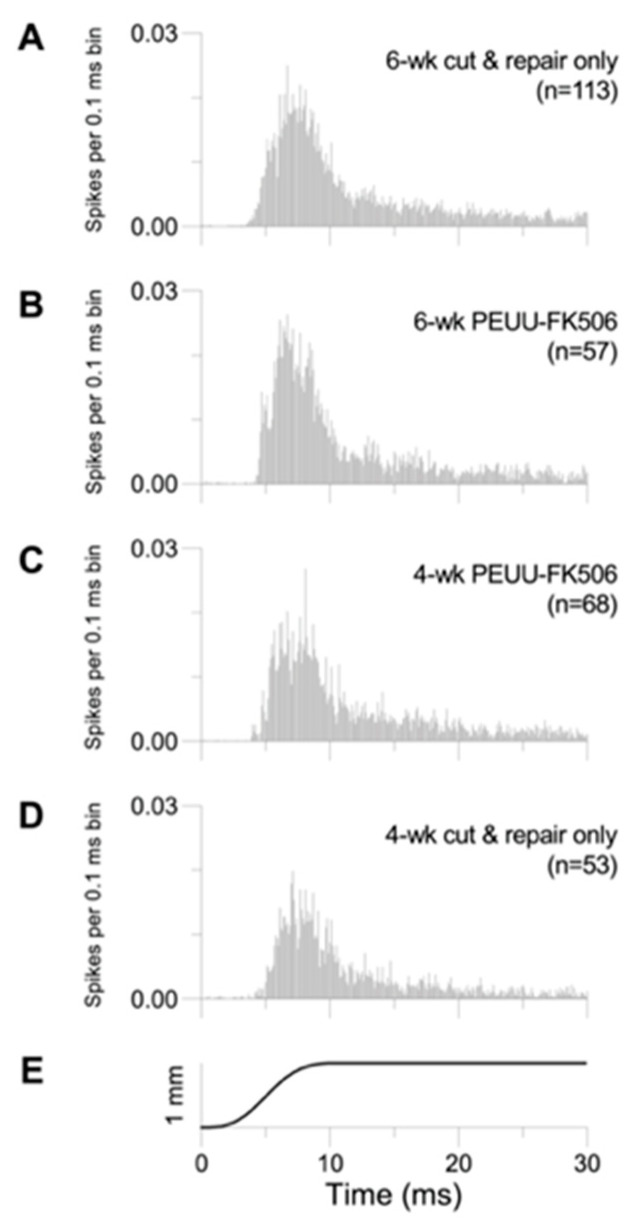
Response magnitude is lower for 4-week recovery cases without PEUU-FK506 treatment. Representative peristimulus time histograms of trigeminal ganglion cell to whisker deflection among treatment groups (**A**–**D**). Each peri-stimulus time histogram consists of *n* = 300 (0.1 ms) bins and is constructed from responses of all neurons within each experimental group (10 deflections × 8 directions × number of neurons; *n* = number of cells); histograms are scaled to the total number of stimuli. Stimulus waveform for movement onsets is shown in panel (**E**).

The effects of the different treatments on trigeminal ganglion cell responses to whisker deflection are illustrated qualitatively by population PSTHs in Figure 2. Shown are spike counts for all deflections accumulated in successive 0.1 ms bins for a period of 30 ms following movement onset which occurs at time 0; the height of each bin is scaled to the total number of stimuli (number of animals x 8 angles x 10 repetitions). ANOVAs for each experimental group indicated that there are no differences related to individual animal subjects; therefore, data are pooled for all recorded cells. In all groups (as in untreated animals [53]), responses are highly transient to the initial movement, display similar time courses, and diminish to low-level firing when the whisker is held in a fixed, deflected position (Figure 2A–D). Overall, the recovery of response magnitude is noticeably smaller for four-week cut-and-repair-only animals (Figure 2D).

**Figure 3:**
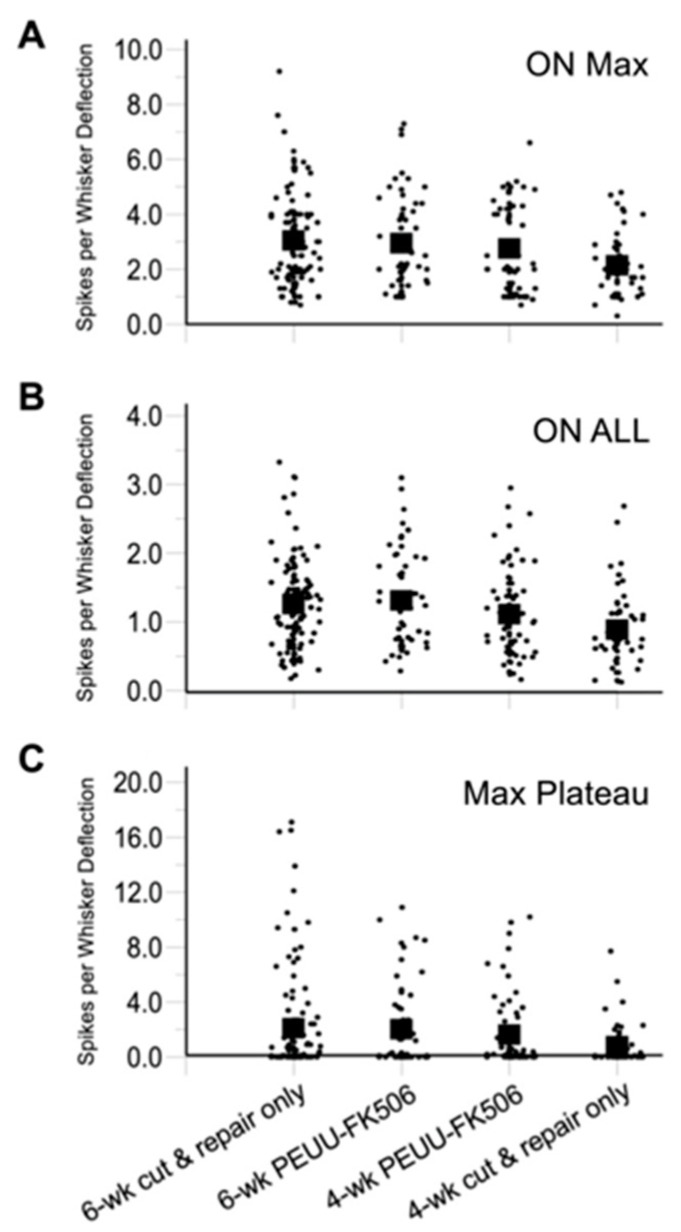
PEUU-FK506 treatment accelerates recovery but does not lead to improved long-term outcomes. Trigeminal ganglion cell to whisker deflection response magnitudes (**A**–**C**). ON responses are calculated as average spikes per stimulus evoked during the first 20 ms of the response following deflection onset. (**A**) ON responses evoked by movement at each cell’s maximally effective deflection angle. (**B**) ON responses averaged over all 8 deflection angles. (**C**) Maximal angle plateau responses calculated over 100 ms. Data for individual neurons within the 4 experimental groups are represented as discrete points. Population mean per group are represented as filled square blocks. Number of cells per group are reported in Table 1.

Data were quantified as average spikes per stimulus evoked during the first 20 ms of the response, i.e., ON responses (Table 1 and Figure 3). For deflections at each cell’s maximally effective direction (“Max Angle”, Figure 3A), the six-week PEUU-FK506 units were found to respond with the same overall firing rates as control six-week cut-and-repair-only firing rates (Dunnett’s test, *p* = 0.97). Thus, PEUU-FK506 treatment did not increase the functional outcome six weeks after nerve transection and repair. Interestingly, four-week PEUU-FK506 animals were as responsive to whisker deflections as six-week cut-and-repair-only animals (*p* = 0.49), whereas untreated four-week cut-and-repair-only animals were less responsive (*p* = 0.001). Similar results were obtained when ON responses were averaged over all eight deflection angles and for plateau responses (Table 1). Taken together, the findings indicate that PEUU-FK506 treatment accelerated the recovery from ION transection but did not improve longer-term functional outcomes.

Table 1 PEUU-FK506 treatment accelerates the recovery process of transected infraorbital nerves. The trigeminal ganglion cell to whisker deflection response magnitudes shown as ON responses per stimulus evoked during the first 20 or 100 ms of the response. *p*-values were calculated in relation to the six-week cut-and-repair-only group by Dunnett’s test with a significance level of *p* < 0.05.

### 2.3. Infraorbital Nerve Immunohistochemistry

**Figure 4:**
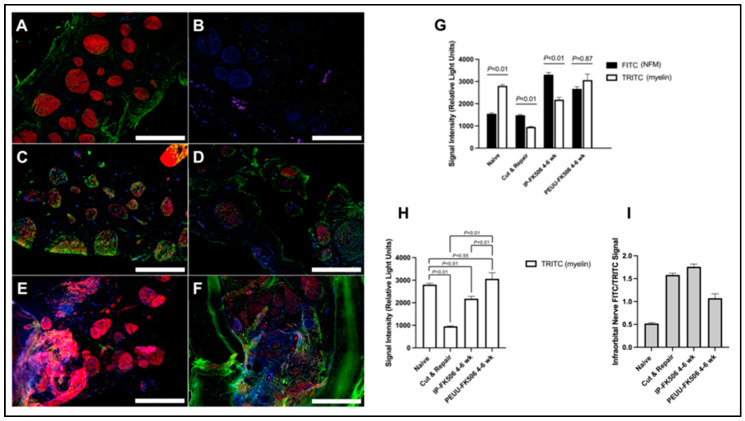
Myelin staining intensity is greater in PEUU-FK506-treated infraorbital nerves versus IP-FK506 treatment. (**A**–**F**) Representative confocal images of infraorbital nerves stained for myelin (red/TRITC), neurofilament medium chain (green/FITC), and nucleus (blue/DAPI). Confocal images acquired for each sample were modified using NIS-Elements software version 4.4.0. Scale bar = 50 microns. (**A**) Naїve; (**B**) cut-and-repair; (**C**) IP-FK506 4-wk; (**D**) IP-FK506 6-wk; (**E**) PEUU-FK506 wrap 4-wk; and (**F**) PEUU-FK506 wrap 6-wk. (**G**) Measured neurofilament medium chain (FITC) and myelin (TRITC) signal within each study cohort (*n* = 2–4 per group). (**H**) Comparison of myelin (TRITC) signal within each study cohort (*n* = 2–4 per group). (**I**) Proportion of infraorbital nerve FITC per TRITC signal within each study cohort (*n* = 2–4 per group). Signal intensity represented as mean ± SEM with significance denoted as *p* < 0.05.

Myelination in the proximal stump was greater in PEUU-FK506-treated IONs compared to IP-FK506 treatment up to six weeks following transection and immediate neurorrhaphy (Figure 4A–I). In the ION of naïve animals, the myelin expression was greater compared to the neurofilament medium chain (NFM) (*p* < 0.01) (Figure 4G). Both cut-and-repair and IP-FK506 groups exhibited a greater NFM expression compared to myelin (*p* < 0.01) and a reduced myelin signal as compared to the naïve controls (*p* < 0.01) (Figure 4G–I). Conversely, PEUU-FK506 treatment increased the ratio of myelin:NFM in the ION, albeit not significantly. With PEUU-FK506 treatment, Schwann cell myelin expression was higher compared against both cut-and-repair and IP-FK506 treatments (*p* < 0.01), but this increase was not significant when compared to the naïve controls (*p* = 0.55) (Figure 4H).

## 3. Discussion

Given the known issues of toxicity associated with systemic FK506 treatment [55], this study investigated whether PEUU-FK506 wraps could locally enrich dedicated sensory peripheral nervous tissue with FK506 while minimizing accumulation within the systemic circulation. Local drug delivery is superior to systemic administration because lower drug dosages are delivered with greater precision to the affected tissues over a desired period. Effective local drug delivery systems can minimize off-target side effects, attenuate metabolism or clearance, reduce administration frequency, and improve patient compliance. Such features are especially important in inflammatory settings [56].

To confirm the local delivery of FK506 by PEUU-FK506, the concentration of FK506 in the systemic circulation and tissues proximal and distal to the transection and repair site were measured using HPLC-tandem mass spectrometry. We found that the levels of FK506 within the ION when delivered using the PEUU-FK506 wrap was 20,461.66 ng/mL (20.46 mg/kg). Meanwhile, daily administration of 2.2 mg/kg (IP) over 14 days enriched the ION FK506 concentration to 195.73 ± 25.43 ng/mL, or approximately ~0.2 mg/kg, representing a 100-fold difference in tissue-specific enrichment of FK506 within the ION. This demonstrates a 100-fold increase in drug delivery to the target tissue, while using twenty-fold less of the total administered drug (~0.4 mg with PEUU-FK506 treatment vs. 7.7 mg with IP-FK506 treatment) over a specific time of 14 days. Regardless of treatment modality, systemic FK506 was either below or nearly below the typical therapeutic trough levels, which range from 10–20 ng/mL. The results are a clear demonstration of the superiority and potential of engineered local drug delivery systems.

The optimal FK506 dose varies among previous reports. Confounders include the type of animal injury model and modality of administration, as well as whether FK506 is used for its neuroregenerative or immunosuppressive properties. Yang et al. report that immunosuppressive doses of 2.0 mg/kg were necessary to prevent skin allograft rejection while nerve regeneration was augmented with sub-immunosuppressive FK506 doses of 0.5 mg/kg and 1.0 mg/kg [57]. Additionally, using a sciatic nerve crush model in Sprague–Dawley rats, Wang et al. found 5.0 mg/kg administered subcutaneously to be optimal for neuroregeneration [58]. Udina et al. used a mouse sciatic nerve crush model to report greatest axonal regeneration at a FK506 dose of 5.0 mg/kg per day [59]. For these studies, the enrichment of FK506 at the nerve is most likely less than the injection concentration due to the combined effects of drug dilution and global distribution. Indeed, our study shows that much higher levels of FK506 within the nerve itself are beneficial to nerve regeneration than previously thought [59]. Future studies with the PEUU-FK506 wrap should aim to elucidate what FK506 concentration within the wrap results in the largest increase in neuroregenerative capacity. As this is the first study to evaluate such a modality in a purely sensory nerve, we aimed to uncover any benefits of using our PEUU-FK506 nerve wrap to treat ION transection.

Previous research has uncovered some of the molecular mechanisms that underly the neurotrophic effect FK506 exerts on peripheral nerve regeneration, including an FK506 interaction with axons at the level of the growth cone [60,61,62]. Importantly, FK506 has been shown to stimulate Schwann cell proliferation in culture and to decrease myelin debris within the distal stump of transected nerves [23,24]. Myelinated axons are critical to the fast signaling found in the nervous system of healthy mammals. In the CNS, oligodendrocytes myelinate multiple axons, while Schwann cells (SCs) myelinate a single axon in the PNS. Recently, Harty et al. demonstrated that the mutation of Fbxw7 in Schwann cells permitted them to unsheathe multiple axons. They suggest that the difference in the myelinating cell: axon ratio between PNS and CNS could account for the limited response to injury in the CNS, while Schwann cells can rapidly and faithfully respond to injuries in the PNS [63]. Therefore, the response to injury by both the neuron and SCs of the PNS can be evaluated by measurements of the axon to myelin ratio dynamics. In this study, we immunolabelled both NFM and myelin in IONs recovered from the various treatment groups and found a greater myelin expression in PEUU-FK506-treated IONs compared to traditional IP-FK506 treatment. This comports with previous demonstrations of enhanced myelinated fiber density in FK506-treated animals at seven weeks after peripheral nerve grafting [23]. These results suggest that PEUU-FK506-treatment-enhanced Schwann cell myelin expression is associated with positive functional outcomes in our study.

While peripheral nerves can regenerate after injury, maximal functional recovery with surgical neurorrhaphy alone is limited without additional rehabilitation [4,7,64,65]. In this scenario, the degree of functional recovery is achieved as a function of time and specific character or neuronal unit response. For example, we previously demonstrated that the six-week responses of slowly adapting cells (SA) are smaller than those after eight weeks of recovery [53]. Accordingly, maximal recovery of the whisker-evoked response was found to be achieved at six weeks in both groups tested in this study. Importantly, we observed that PEUU-FK506 treatment accelerated the functional recovery of the whisker-evoked trigeminal neuron responses (Figure 3). At four weeks postoperatively, PEUU-FK506-treated animals had already achieved functional recovery as measured by evoked responses comparable to those of the six-week rapidly adapting cells (RA). While we did not distinguish between RAs and SAs, the results of this study suggest that FK506 is most beneficial at the early stages of recovery, and, therefore, such treatment should be initiated as quickly as possible following injury. The choice not to distinguish between RA and SA responses limits this study; however, our principal conclusion agrees with general medical treatment and also improved nerve repair outcomes with early treatment after injury [66]. Other limitations of this study include the decision to record from the trigeminal ganglion which is a mixed sensory and motor nerve. In humans, functional outcome following sensory nerve repair worsens with time. Future work could include discrete cortical recordings from either the primary somatosensory or motor cortex to investigate possible differences in FK506 wrap treatment on neuron type. Another possible experiment could investigate whether accelerated two-point discrimination recovery following nerve injury might result with FK506 nerve wrap treatment. Further, sex differences in the outcomes of PNI have been reported, where female rats appear more susceptible to the development of spread mechanical hypersensitivity, compared with male rats [67]. The potential for sex differences in this study were not investigated but should be undertaken in future work. Regardless of these limitations, the benefits of earlier functional recovery following PNI is clearly clinically meaningful to patients and their quality of life.

The potentially fatal side effects of systemic immunosuppression in composite tissue allotransplantation such as infection and malignancy have severely limited its scope in practice. As such, many studies are investigating ways to avoid systemic immunosuppression while still inhibiting the rejection of the transplanted tissue [15]. Additionally, there is at least one account of composite allograft rejection due to medication non-adherence [68]. All this engenders great promise for identifying a method of local immunosuppression that is not dependent on patient compliance. In this study, we showed that the target nerve was highly enriched in FK506, whereas the systemic blood level was nearly negligible with a maximum measurement at 2.93 ng/mL. The levels of FK506 associated with nephrotoxicity range from 14.5–50.5 ng/mL [69]. This is a promising result for the possibility of using localized FK506 in VCA as both an immunosuppressant and an adjuvant therapy to promote neuroregeneration and improve functional outcomes.

## 4. Materials and Methods

### 4.1. Animals

This study used 8–10-week-old male Lewis (RT1) rats (Charles River Laboratories, Inc., Wilmington, MA, USA) with weights ranging from 290–350 g. Animals were matched based on strain, sex, and age to maintain homogeneity of characteristics between cohorts. All animals received care in compliance with the Institutional Animal Care and Use Committee and protocol approval from the University of Pittsburgh and followed guidelines from the *Guide for the Care and Use of Laboratory Animals* published by the U.S. National Institutes of Health (Bethesda, MD, USA).

### 4.2. Animals for FK506 Concentration Measurements

**Figure 5:**
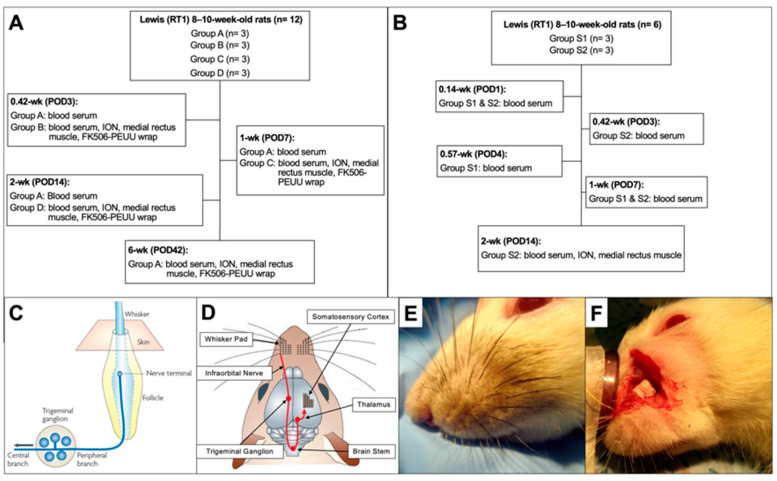
Flowchart of cohorts assigned to FK506 concentration measurements in the blood serum, ION, medial rectus muscle, and PEUU-FK506 at designated timepoints. (**A**) Animal cohorts assigned to PEUU-FK506 treatment. (**B**) Animal cohorts assigned to IP-FK506 treatment. (**C**) Representative diagram of whisker/trigeminal system model for recording trigeminal nerve nuclei signal. (**D**) Spatial representation of anatomical structures involved in measuring mechanoreceptive afferent signal. (**E**) Naïve rat with intact ION (no surgery). (**F**) ION transection and repair with PEUU-FK506 treatment.

Eighteen 8–10-week-old Lewis (RT1) rats underwent surgery to expose the ION (*n* = 12 in PEUU-FK506 group, *n* = 6 in systemic FK506 group). Subjects in the PEUU-FK506 group received a 5 mm × 1.5 mm section of 10 mg PEUU-FK506 wrap around the ION whereas systemic FK506 subjects were injected daily with 2.2 mg/kg/day FK506 intraperitoneally (hereafter denoted as “IP-FK506”). In the PEUU-FK506-treated group, FK506 levels were measured in the blood serum, ION, medial rectus muscle, and recovered nerve wrap up to six weeks postoperatively (Figure 5A). In the IP-FK506-treated group, blood serum measurements were taken up to two weeks postoperatively (Figure 5B). FK506 concentration in the ION and medial rectus muscle were recorded at the 2-week (POD14) timepoint for IP-FK506-treated subjects. Detailed characterization of the PEUU-FK506 wrap can be found below and are also previously reported [50,70].

### 4.3. Animals for Trigeminal Ganglion Cell Recordings

Two groups were created from 24 total 8–10-week-old Lewis (RT1) rats to measure response at four weeks and six weeks after ION transection and repair: (1) PEUU-FK506 treatment group (*n* = 3–6/group/timepoint) and (2) neurorrhaphy-only non-treatment group (*n* = 5–10/group/timepoint). PEUU-FK506 treatment animals received PEUU-FK506 wrap (as above) in addition to neurorrhaphy after ION transection.

### 4.4. Infraorbital Nerve Transection and Repair

Surgery animals were anesthetized with 50 mg/kg intraperitoneal pentobarbital sodium and supplemented as necessary with 10 mg/kg injections. Sterile technique was utilized for all surgeries and a servo-controlled heating blanket (Harvard Apparatus, Cambridge, MA, USA) was used to maintain core animal temperature at 37 °C. The surgical procedure was performed as previously described [53]. Briefly, proximal to the mystacial pad, an incision was made on the left hemiface, distal to where the infraorbital nerve exits from the infraorbital foramen. Tissue was discretely dissected to expose the infraorbital branch of the trigeminal nerve and was then transected using micro-scissors. Following transection, the nerve trunks were coapted using standard microsurgical nerve repair procedure with 10-0 nylon suture using approximately 10 sutures. Following coaptation of the nerve in the treatment group, a 5 mm × 1.5 mm section of 10 mg PEUU-FK506 wrap was sutured to itself around the transection and repair portion of the nerve. Finally, the skin incision was closed with 5-0 chromic dissolvable sutures. The rats were observed until recovery from anesthesia and returned to individual cages. Rats were given a standard 12 h light–dark cycle with rat chow and water available ad libitum.

### 4.5. PEUU-FK506 Sheet Fabrication

PEUU was synthesized using a two-step solution polymerization from polycaprolactone and 1,4-diisocyanatobutane. Putrescine was used as chain extender [71]. Sheets containing FK506 were fabricated by electrospinning [72]. The synthesis of PEUU-FK506 sheets has been previously described in detail [50]. Briefly, 20 mg of FK506 was dissolved in DMSO to create a 20 mM solution. This FK506 solution was mixed with 0.45 g PEUU, which was dissolved in 1,1,1,3,3,3-hexafluoroisopropanol (HFIP) at a concentration of 12% (*w*/*v*), and electrospun onto a rotating stainless-steel mandrel (17 cm from capillary tip) by feeding through a charged capillary at a rate of 1.5 mL/h. Voltage between the capillary and mandrel was 19 kV. The 0.1 mm thick sheet with total volume 370 mm^3^ was sterilized under UV light overnight and then with EtOH prior to surgical implantation.

### 4.6. Structural and Mechanical Properties of PEUU-FK506 Sheet

A tacrolimus-releasing matrix was constructed by extending a previously reported electrospinning protocol for PEUU [70]. Macroscopically, both unloaded PEUU and the PEUU-FK506 matrices exhibited an off-white, pliable appearance and were indistinguishable from each other [50]. The smooth surface characteristics were maintained, contributing to the desirable features of the matrix in the context of CNS nerve applications. Scanning electron microscopy (SEM) was conducted to investigate the electrospun fibers in both PEUU and PEUU-FK506 matrices. The analysis revealed randomly organized polymer fibers with varied diameters in both matrices. Notably, the average polymer fiber diameter remained consistent across all matrices, with unloaded PEUU fibers measuring 510 ± 130 nm, and 10 or 20 mg loaded PEUU-FK506 fibers having average diameters of 560 ± 210 nm and 560 ± 160 nm, respectively.

Evaluation of mechanical properties [50] included the impact of FK506 loading on Young’s modulus, strain at break, and ultimate stress. Tacrolimus loading did not significantly alter Young’s modulus, indicating values of 25 ± 9 MPa for PEUU, 19 ± 12 MPa for 10 mg PEUU-FK506, and 15 ± 11 MPa for 20 mg PEUU-FK506. Strain at break exhibited no significant differences, with values of 262 ± 21% for PEUU, 217 ± 67% for 10 mg PEUU-FK506, and 222 ± 34% for 20 mg PEUU-FK506. However, ultimate stress decreased significantly in tacrolimus-loaded matrices compared to PEUU, measuring 13 ± 4 MPa for PEUU, 6 ± 0.5 MPa for 10 mg PEUU-Tac, and 6 ± 3 MPa for 20 mg PEUU-FK506.

We previously assessed the surface properties of the electrospun PEUU and tacrolimus elastomeric matrix. Through zeta potential analysis, we characterized the surface charge, revealing a slightly negative charge that has potential implications for cellular interactions. Fourier-transform infrared spectroscopy (FTIR) elucidated specific functional groups, such as ester and urea groups in the polymer structure. These surface properties play a vital role in influencing cellular adhesion and overall biocompatibility, enhancing the potential for effective interactions with biological tissues in drug delivery applications.

The safety and biocompatibility of PEUU-FK506 were also rigorously evaluated using cell culture and in vivo assessments in rat model of nerve injury [50]. In this study targeted at the infraorbital nerve, careful observations for any signs of adverse reactions or inflammation were conducted, and, notably, no overt signs of necrosis or inflammation were observed in any of the animals subjected to the PEUU and tacrolimus elastomeric matrix. Overall, these structural and mechanical analyses affirm the consistency of the drug carrier’s properties crucial for in vivo performance.

### 4.7. Extraction of FK506 from Tissue and Wrap

Infraorbital nerve, medial rectus muscle, and/or PEUU-FK506 wrap were discretely dissected from the 18 animals assigned for FK506 concentration analysis as described above, then weighed prior to homogenization with methanol (100%) using Mini-BeadBeater-1 (BioSpec, Bartlesville, OK, USA) for cell or scaffold disruption. The homogenate was left overnight in a sonicator to allow for the complete extraction of the drug from the material. The recovered homogenate was centrifuged at 2100 ± 100 rpm for 10 min. Supernatant was evaporated by a sample concentrator, and the drug residue was reconstituted with blood.

### 4.8. Quantification of FK506 by LC-Tandem Mass Spectrometry (LC-MS/MS)

To precipitate plasma proteins, 50 µL of blood containing an unknown concentration of FK506 was combined with 200 µL of zinc sulfate heptahydrate (ZnSO_4_·7H_2_O) in a conical centrifuge tube. Then, 500 µL of an acetonitrile-based solution containing a deuterated internal standard (ascomycin at a concentration of 15 ng/mL) was added to the mixture followed by vortexing at 3000 rpm for 2 min, and then centrifuged at 13,000 rpm for 3 min. The supernatants were collected in LCMS vials for analysis. As described previously [50], sample analysis was performed using a fully validated, reverse-phased high-performance liquid chromatographic method for detecting tacrolimus on a Waters micromass Quattro micro-API mass spectrometer (Waters, Milford, MA, USA) that is operated in positive electrospray ionization mode, utilizing multiple reaction monitoring, with an injection volume of 20 µL. The Waters 2795 Alliance Separations Module was equipped with a Nova-Pak^®^ C18 column, 2.1 × 10 mm cartridge (Waters #186003523, Milford, MA, USA) heated to 55 °C. Analytes were effectively separated using a gradient elution consisting of an aqueous mobile phase (95% H_2_O/5% MeOH) and an organic mobile phase (100% MeOH), at a flow rate of 0.6 mL per minute. Mobile phases also contained 0.1% formic acid (CH_2_O_2_) and 2 mM ammonium acetate. Statistical analysis was conducted using Student *t*-tests for significance.

### 4.9. Trigeminal Ganglion Cell Recordings

Responses of individual trigeminal ganglion neurons were recorded as previously described [53,54]. Briefly, rats were anesthetized with 1–2% isoflurane in a 1:1 mixture of N_2_ and O_2_. A servo-controlled heating pad was used to maintain core temperature at 37 °C. Polyethylene tubing was inserted into the trachea as a cannula, the dorsal surface of the skull was exposed, and a steel post fixed to it with dental acrylic, allowing unimpeded access to the left facial whiskers. For electrical grounding, a stainless-steel screw was inserted into the frontal region of the animal’s skull. A dental drill was used to create a left-sided craniotomy overlying the left trigeminal ganglion at the skull base approximately 10 mm below the cortical surface. Neural recordings lasted ~10 h after which the animal was euthanized with an overdose of isoflurane.

Electrophysiological signals were amplified and filtered at 0.3–10 kHz using a Grass pre-amplifier. Single-cell or “unit” activity was monitored aurally using a noise-clipping circuit and visualized by oscilloscope. Varnish-coated stainless steel or tungsten microelectrodes (2–4 MW at 1000 Hz (FHC, Bowdoin, ME, USA)) were advanced from the brain’s dorsal surface into the left trigeminal ganglion using a hydraulic microdrive. As the microelectrode was advanced, whiskers were deflected using hand-held probes; at each responsive site, the whisker evoking the most robust and consistent response was identified as the Principal Whisker (PW) [52,73]. All whiskers were trimmed to ~12 mm. To identify individual whiskers, the face was observed using a stereoscope. Evoked somatosensory responses were identified as short-latency activity that could be evoked by gentle manual deflection of the whiskers. Evoked responses were quantified recorded by deflecting the PW in a controlled fashion using a multi-angle piezoelectric stimulator that moved the whisker in 8 directions in 45° increments [74]. Each whisker was attached to the stimulator at 10 mm from the skin surface and moved 1 mm using a ramp-and-hold stimulus of 125 mm/s and held steady at that plateau position for 200 ms prior to return of the whisker to its undeflected or neutral position. Deflections were randomized in 10 blocks of 8 directions for a total of 80 stimulus presentations. A laboratory computer controlled the stimulator and collected the spike times measured at 32 kHz, and spike waveforms were saved to disk for analysis.

### 4.10. Experimental Design and Data Analysis for Trigeminal Ganglion Cell Response

Response data were obtained from four groups of animals, with all receiving surgical nerve transection and repair: (group 1) 4-week post-transection (hereafter denoted as “4-wk cut & repair only”, *n* = 5); (group 2) 4-week post-transection with PEUU-FK506 treatment (“4-wk PEUU-FK506”, *n*= 6); (group 3) 6-week post-transection (“6-wk cut & repair only”, *n* = 10); and (group 4) 6-week post-transection with PEUU-FK506 treatment (“6-wk PEUU-FK506”, *n* = 3). Electrophysiological data were analyzed with Microsoft Excel version 16.8 using custom software written in Visual Basic (Microsoft Visual Studio 2022 version 17.8.1). Responses to whisker movement are transient and brief, followed in some cells by lower levels of sustained discharge during the steady-state stimulus plateau (Figure 2). ON responses were quantified as average spike counts per deflection during the first 20 ms following stimulus onset and during the latter 100 ms of the steady state phase of the stimulus, i.e., the plateau. These counts, expressed as spike per stimulus, were used to identify each cell’s maximally effective or “best” deflection angle; deflections in the other directions evoke fewer or no spikes. ON response data were examined separately for maximal angle deflections (*n* = 10) and for deflections averaged over all directions (*n* = 80). Plateau firing rates are low and often null; therefore, we examined only maximal angle spike counts. Statistical analyses were performed using the Microsoft Excel add-on Analyze-it (Analyze-it Software version 16.5.4, Leeds, UK). Dunnett tests for multiple comparisons examined spike counts for all four groups using the “6-wk cut & repair only” cohort as the control group.

### 4.11. Immunohistochemistry

Myelin and axonal neurofilament staining after ION transection and repair were assessed in animals assigned to neurorrhaphy ± FK506 treatment groups for comparison with naïve animals. Immunofluorescent staining of histological ION samples was performed as previously described [75]. Briefly, animals were euthanized, and IONs were dissected at the 4- and 6-week timepoints postoperatively. Collected nerves were immediately fixed with 4% paraformaldehyde (PFA) in phosphate-buffered saline (PBS) for a minimum time of 2 h. Fixed nerves were then cryoprotected in 30% sucrose in PBS (4–12 h), embedded in optimal cutting temperature medium, and frozen in liquid nitrogen followed by storage at −80 °C prior to sectioning. The ION samples were cut into 15 µm transverse sections anterior to the repair site on a cryostat and transferred to glass slides for immunolabelling. These ION sections were then labeled with anti-neurofilament medium chain (1:400, Thermo Scientific/Pierce, Waltham, MA USA) and anti-myelin (1:300, Life Technologies, Carlsbad, CA, USA), and nuclei were stained with DAPI (Vector Laboratories, Newark, CA, USA). Samples were imaged with confocal microscopy, and fluorescence intensity was measured using ImageJ version 1.53e software.

## 5. Conclusions

The PEUU-FK506 nerve wraps used in this study appeared to significantly accelerate neuroregeneration in a sensory nerve at four weeks after axotomy and repair. Histomorphometric analysis showed greater myelin expression in PEUU-FK506-treated infraorbital nerves up to six weeks after axotomy and repair. Additionally, PEUU-FK506 wraps were able to enrich local tissue with FK506 without spilling over appreciably into the systemic circulation. This modality can be specifically titrated for the tissue of interest and may result in a promising method for nerve regeneration, as well as local immunosuppression in composite tissue allotransplantation.

## Figures and Tables

**Table 1:** ON responses.

Group	ON Max (20 ms)	ON ALL (20 ms)	Max Plateau (100 ms)
6-wk cut & repair only (*n* = 113)	3.05 ± 1.61	1.26 ± 0.65	2.10 ± 3.80
6-wk PEEU-FK506 (*n* = 57)	2.95 ± 1.67; *p* = 0.97	1.30 ± 0.68; *p* = 0.96	1.99 ± 3.07; *p* = 0.99
4-wk PEEU-FK506 (*n* = 68)	2.76 ± 1.51; *p* = 0.49	1.15 ± 0.64; *p* = 0.34	1.62 ± 2.56; *p* = 0.65
4-wk cut & repair only (*n* = 53)	2.15 ± 1.08; *p* = 0.001	0.89 ± 0.55; *p* = 0.002	0.78 ± 1.50; *p* = 0.03

## Data Availability

The data presented in this study are available in the main text or upon request from the corresponding author.
